# Characterization of the complete chloroplast genome of *Senna bicapsularis* (Leguminosae), an ornamental plant

**DOI:** 10.1080/23802359.2020.1782785

**Published:** 2020-07-02

**Authors:** Xueyan Zhao, Fangyuan Wang, Ruirui Zhang, Yan Li

**Affiliations:** a Shaanxi Engineering Research Centre for Conservation and Utilization of Botanical Resources, Xi’an Botanical Garden of Shaanxi Province (Institute of Botany of Shaanxi Province), Xi’an, China; b School of Pharmacy, Shaanxi Institute of International Trade and Commerce, Xianyang, China

**Keywords:** *Senna bicapsularis*, chloroplast genome, phylogenetic analysis

## Abstract

*Senna bicapsularis* is an important flowering, ornamental plant. The extracts showed antioxidant activity and antibacterial properties. In addition, *S. bicapsularis* can be used as a model woody plants. In the present study, the complete chloroplast genome was sequenced. The result showed that the length of circular chloroplast genome was 162,744 bp, containing a large single-copy region of 91,176 bp, a small single-copy region of 18,264 bp, and two inverted repeat regions of 26,652 bp. The chloroplast genome contained 128 genes, including 83 protein-coding, eight rRNA, and 37 tRNA genes. Phylogenetic tree analysis showed that *S. bicapsularis* has closely relationship with *Senna occidentalis*, *Senna tora* and *Senna siamea*.

*Senna bicapsularis* (L.) Roxb. which belongs to *Senna* (Leguminosae) is one the important flowering, ornamental plant. *Senna* species are widely distributed in South American and tropical countries (Han et al. 2006). *S. bicapsularis* has medicinal values, and the flower extracts showed antioxidant activity and antibacterial properties (Mak et al. 2013). *S. bicapsularis* has strong adaptability and is widely used in the garden. Previous studies have shown that *S. bicapsularis* has cold, heat, waterlogging, and salt resistances (Lu 2007; Liao 2010; Cai and Liao 2017; Liu et al. 2019). *S. bicapsularis* sowed in spring, and bloomed and seeded in the same year. The growth cycle is short and can be used as model woody plants for the study of genetic variation and secondary growth. In the present study, we characterize the complete chloroplast genome of *S. bicapsularis* and provide basic data for studying the phylogenetic relationships in Leguminosae.

The fresh leaves of *S. bicapsularis* were collected from Xi’an Botanical Garden of Shaanxi Province (34°21′N, 108°95′E; Shaanxi, China), and the voucher specimen (ZY190202) was deposited in Xi’an Botanical Garden Herbarium. Chloroplast genomic DNA was extracted from the fresh leaves using the modified CTAB method (Doyle and Doyle 1987). Total DNA was used for the shotgun library construction and the subsequent high-throughput sequencing on the Illumina HiSeq 2500 Sequencing System.

After quality-trimmed, the obtained data were assembled using MITObim v1.8 with the reference sequence of *Senna tora* (GenBank: NC_030193) (Hahn et al. 2013). Whereafter, the genome was annotated using Geneious v9.0.2 (Biomatters Ltd., Auckland, New Zealand) by aligning with the reference chloroplast genome. The circular plastid genome map was completed using the online program OGDRAW (Lohse et al. 2013). The annotated chloroplast genome sequence has been deposited into the NCBI genbank (accession number: MT559309).

The total plastome length of *S. bicapsularis* was 162,744 bp, with large single copy (LSC, 91,176 bp), small single copy (SSC, 18,264 bp), and two inverted repeats (IRa and IRb; 26,652 bp each). The overall GC content was 36.1% (LSC: 33.6%; SSC: 30.0%; IRs: 42.4%) and the chloroplast genome contained 128 genes, including 83 protein-coding, eight rRNA, and 37 tRNA genes.

In order to investigate the phylogenetic relationship of *S. bicapsularis* in Leguminosae, the phylogenetic tree was constructed with MEG6 (Tamura et al. 2013) based on 18 complete chloroplast genome sequences and *Sanguisorba officinalis* (Rosaceae) (GenBank: NC_044694) was used as an outgroup (Figure 1). The results indicated that compared with other genera plants of Leguminosae, *S. bicapsularis* has close relationship with *S. occidentalis*, *S. tora*, and *Senna siamea*.

**Figure 1.**
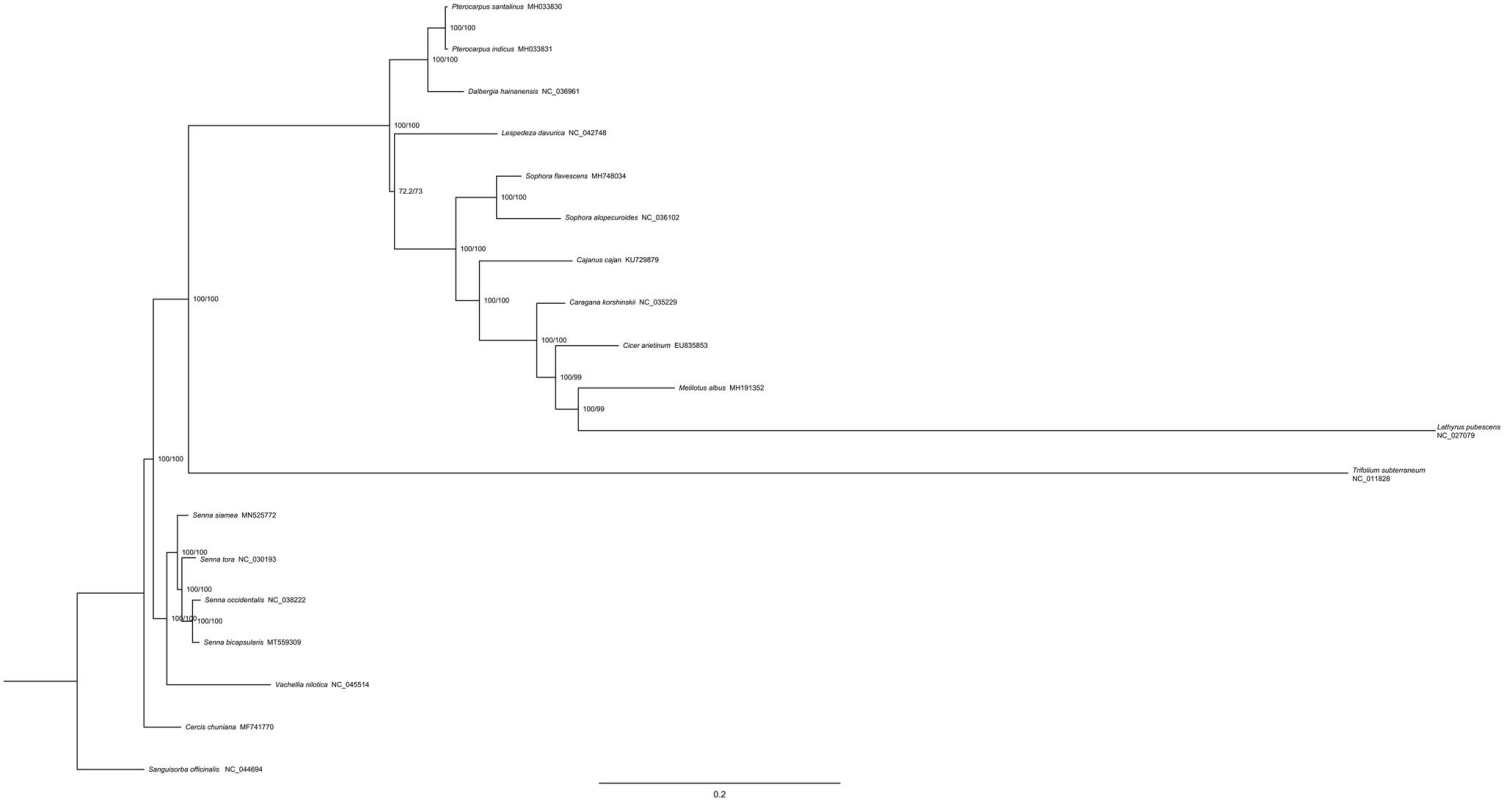
Phylogenetic tree based on 19 complete chloroplast genome sequences in Leguminosae.

## Data Availability

The data that support the findings of this study are openly available in National Center for Biotechnology Information] at [https://www.ncbi.nlm.nih.gov/], accession number [MT559309].

## References

[CIT0001] Cai SQ, Liao FY. 2017. Effects of waterlogging stress on growth and physiological characteristics of *Cassia bicapsularis*. Northern Hortic. 3:71–75.

[CIT0002] Doyle JJ, Doyle JL. 1987. A rapid DNA isolation procedure for small quantities of fresh leaf tissue. Phytochem Bull. 19:11–15.

[CIT0003] Hahn C, Bachmann L, Chevreux B. 2013. Reconstructing mitochondrial genomes directly from genomic next-generation sequencing reads: a baiting and iterative mapping approach. Nucleic Acids Res. 41(13):e129. 23661685 10.1093/nar/gkt371 PMC3711436

[CIT0004] Han S, Shi DX, Wang ML, Liao J, Mai MM. 2006. Tissue culture and plantlet regeneration of *Cassia bicapsularis* Linn. Plant Physiol Commun. 24(5):245.

[CIT0005] Liao FY. 2010. The effect of high temperature and intensity light on the physio-ecological indexs of *Cassia bicapsularis* and its application in the landscape and architecture. Northern Hortic. 7:96–99.

[CIT0006] Liu GY, Wang Q, Mao ZX. 2019. Effects of natural low temperature on cold-resistance physiological indexes of three subtropical ornamental plants. Mol Plant Breeding. 17(15):5136–5143.

[CIT0007] Lohse M, Drechsel O, Kahlau S, Bock R. 2013. Organellar Genome DRAW – a suite of tools for generating physical maps of plastid and mitochondrial genomes and visualizing expression data sets. Nucleic Acids Res. 41(Web Server issue):W575–W581. 23609545 10.1093/nar/gkt289 PMC3692101

[CIT0008] Lu F. 2007. Determination of the cold-resistance of *Cassia bicapsularis* and *C. corymbosa* by electric conductivity method. J Central South Univ Forest Technol. 27(3):84–86.

[CIT0009] Mak YW, Chuah LO, Ahmad R, Bhat R. 2013. Antioxidant and antibacterial activities of hibiscus (*Hibiscus rosa-sinensis* L.) and *Cassia* (*Senna bicapsularis* L.) flower extracts. J King Saud Univ Sci. 25(4):275–282.

[CIT0010] Tamura K, Stecher G, Peterson D, Filipski A, Kumar S. 2013. MEGA6: molecular evolutionary genetics analysis version 6.0. Mol Biol Evol. 30(12):2725–2729. 24132122 10.1093/molbev/mst197 PMC3840312

